# Nitrate and oxygen significantly changed the abundance rather than structure of sulphate‐reducing and sulphur‐oxidising bacteria in water retrieved from petroleum reservoirs

**DOI:** 10.1111/1758-2229.13248

**Published:** 2024-04-05

**Authors:** Huimei Tian, Peike Gao, Chen Qi, Guoqiang Li, Ting Ma

**Affiliations:** ^1^ College of Forestry Shandong Agricultural University Taian China; ^2^ Ecology Postdoctoral Mobile Station Forestry College of Shandong Agricultural University Taian China; ^3^ College of Life Sciences Qufu Normal University Jining China; ^4^ Key Laboratory of Molecular Microbiology and Technology, Ministry of Education, College of Life Sciences Nankai University Tianjin China

## Abstract

Sulphate‐reducing bacteria (SRB) are the main culprits of microbiologically influenced corrosion in water‐flooding petroleum reservoirs, but some sulphur‐oxidising bacteria (SOB) are stimulated when nitrate and oxygen are injected, which control the growth of SRB. This study aimed to determine the distributions of SRB and SOB communities in injection–production systems and to analyse the responses of these bacteria to different treatments involving nitrate and oxygen. *Desulfovibrio*, *Desulfobacca*, *Desulfobulbus*, *Sulfuricurvum* and *Dechloromonas* were commonly detected via 16S rRNA gene sequencing. Still, no significant differences were observed for either the SRB or SOB communities between injection and production wells. Three groups of water samples collected from different sampling sites were incubated. Statistical analysis of functional gene (*dsrB* and *soxB*) clone libraries and quantitative polymerase chain reaction showed that the SOB community structures were more strongly affected by the nitrate and oxygen levels than SRB clustered according to the sampling site; moreover, both the SRB and SOB community abundances significantly changed. Additionally, the highest SRB inhibitory effect and the lowest *dsrB/soxB* ratio were obtained under high concentrations of nitrate and oxygen in the three groups, suggesting that the synergistic effect of nitrate and oxygen level was strong on the inhibition of SRB by potential SOB.

## Graphical abstract

Both sulphate‐reducing bacteria (SRB) and sulphur‐oxidising bacteria (SOB) communities showed similarities in the injection and production system. SOB community assembly was more affected by nitrate and oxygen than the SRB community. Nitrate and oxygen changed the abundance of functional genes and their inhibitory effect.

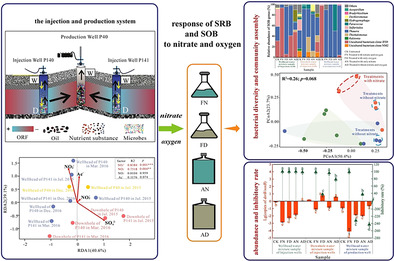

## INTRODUCTION

Water flooding is widely employed in industrial petroleum reservoirs as an efficient and inexpensive oil recovery process known as secondary recovery. In this process, water is injected into oil reservoirs to increase pressure, and the oil is swept to increase oil production. Along with other sources of water (often seawater), production water is reinjected into injection wells through a series of treatments, for example, distillation, to remove harmful microorganisms such as sulphate‐reducing bacteria (SRB), thus forming an injection and production system. Although oil recovery has improved with the development of water‐flooding technology, several adverse effects have been observed in petroleum reservoirs, such as metal corrosion and souring (Gieg et al., 2011; Hubert et al., 2005; Johnson et al., 2017; Okpala & Voordouw, 2018; Qi et al., 2021, 2022; Williamson et al., 2020). Microorganisms inhabiting petroleum reservoirs, such as SRB, thiosulphate‐reducing bacteria, nitrate‐reducing bacteria (NRB), acetogenic bacteria and methanogenic archaea, play crucial roles in the occurrence of corrosion (Lahme et al., 2021; Li et al., 2016; Marietou, 2021; Rajbongshi & Gogoi, 2021; Vigneron et al., 2016).

SRB are considered the main culprits of microbiologically influenced corrosion (MIC); these bacteria are commonly detected in reservoir environments and exhibit a high level of diversity (Enning & Garrelfs, 2014; Gieg et al., 2011; Gu et al., 2009, 2019; Marietou, 2021; Zhou et al., 2023). Since von Wolzogen Kühr and van der Vlugt identified SRB as the prime cause of widespread iron pipe failures in the sulphate‐rich soils of North Holland in 1934, these bacteria have attracted substantial attention (Dutta et al., 2020; Marietou, 2021; Santos et al., 2022; Tiburcio et al., 2021; von Wolzogen Kuhr & van der Vlugt, 1934). The high concentrations of sulphate (28 mM) introduced via seawater and labile volatile fatty acids with crude oil components (e.g., acetate, propionate and butyrate) in reservoirs provide an ideal environment for the activity of SRB (Gieg et al., 2011). Therefore, SRB can proliferate and cause equipment corrosion, oil extraction pipeline plugging, oil quality reduction and even damage to human health. Coupled with dissimilatory sulphate reduction, SRB catalyse the cycling of carbon (including aliphatic and aromatic hydrocarbons), nitrogen (nitrate and nitrite) and various metals (uranium) (Agrawal et al., 2012; Aoyagi et al., 2021; Greene et al., 2003; Zhou et al., 2011). Enning and Garrelfs (2014) reported that some SRB damage iron structures through the production of hydrogen sulphide, a corrosive chemical agent, and certain SRB can also attack iron structures via electron withdrawal through direct metabolic coupling. Recent studies have revealed that endogenous hydrogen sulphide produced by SRB is not the primary factor causing corrosion; instead, SRB‐induced pipeline corrosion is closely related to extracellular electron transfer (Gu et al., 2019; Jia et al., 2018; Xu et al., 2023). Moreover, the presence of hydrogenases, particularly in Gram‐negative bacteria, enables many SRB to efficiently catalyse hydrogen metabolism and generate energy for sulphate reduction. Thus, H_2_‐utilising bacteria are considered to accelerate corrosion (e.g., *Desulfovibrio* and *Desulfomicrobium*) since H_2_ can serve as an important electron carrier that shuttles electrons from Fe(0) to microorganisms (Li et al., 2018; Philips, 2020; Tang et al., 2019).

To avoid hydrogen sulphide formation in oil fields, the injection of oxygen‐containing nitrate solutions is a widely used measure for the petroleum industry to stimulate NRB to compete with SRB for degradable organics in oil, oxidise hydrogen sulphide to sulphur or sulphate, and suppress sulphate reduction by nitrite or by the higher redox potential that is created by nitrate and oxygen injection (Bødtker et al., 2008; Hubert & Voordouw, 2007; Jurelevicius et al., 2020; Kaster et al., 2007; Marietou et al., 2020; Qi et al., 2022; Voordouw et al., 2009). Studies have also shown that many sulphur‐oxidising bacteria (SOB), which can catalyse sulphide oxidation coupled with nitrate reduction, are stimulated by nitrate (Aoyagi et al., 2021; Hubert & Voordouw, 2007; Jahanbani Veshareh & Nick, 2019; Qi et al., 2022). As reservoir environments contain numerous reduced sulphur compounds (e.g., thiosulphate, sulphide and ferrous sulphide and elemental sulphur), SOB, including *Thiobacillus*, *Sulfurospirillum*, *Sulfuricurvum*, *Sulfurovum* and *Sulfurimonas*, are commonly detected in reservoir environments (Gao et al., 2015, 2022; Gao & Fan, 2023; Hubbard et al., 2014; Tian et al., 2017). The phylogeny of SOB is highly diverse, and they can catalyse sulphur oxidation coupled with carbon and nitrogen cycling under both aerobic and anaerobic conditions (Friedrich, 1998; Ghosh & Dam, 2009). Jahanbani Veshareh and Nick (2019) reported that nitrite inhibition by nitrate‐reducing sulphur‐oxidising bacteria (NR‐SOB) is a significant contributor to nitrate treatment and that the sulphide oxidisation rate by NR‐SOB is equal to or greater than that of SRB. Qi et al. (2021) isolated a potential bioaugmenter, *Gordonia* sp. TD‐4, a dissimilatory nitrate reduction to ammonium (DNRA) driven SOB with good nitrite accumulation performance and a high potential for souring control, to control biosafety. Subsequently, they confirmed that pulsed nitrate addition combined with bioaugmentation with TD‐4 after souring could effectively control souring and enhance the effective duration of nitrate‐mediated souring control as the interactions between SRB and NRB were regulated during the process (Qi et al., 2022). Hence, attention should also be given to the SOB community assembly after nitrate injection into oil reservoirs due to their potential role in corrosion control.

To our knowledge, most of the attention has been given to the distribution of SRB in production wells (Li et al., 2016, 2017; Tian et al., 2017; Tiburcio et al., 2021; Zhou et al., 2023), but relevant studies in injection wells are limited. Additionally, previous studies have shown that the microbial populations present in injected water can pass through reservoir strata, reach production wells and affect the microbial community of production wells to a certain extent (Gao et al., 2015, 2022; Ren et al., 2015). Therefore, it is essential to explore the composition of SRB and SOB in injection–production systems. Here, we aimed to study (1) the differences in the compositions of sulphate‐reducing and sulphur‐oxidising microorganisms in water‐flooded petroleum reservoirs at different sampling sites and during different periods in an injection–production system; (2) the effects of nitrate and oxygen on the distribution and abundance of SRB and SOB in water samples retrieved from different sites in an injection–production system; and (3) the rate of inhibition of SRB by nitrate and oxygen. To achieve the first objective, 16S rRNA gene sequencing was employed. For the second and third objectives, laboratory incubation experiments were conducted on three water samples under four treatments with different concentrations of nitrate and oxygen. The *dsrB* and *soxB* genes encode major enzymes involved in sulphate reduction and sulphur oxidation, and they have been used as phylogenetic markers for the identification of SRB and SOB in diverse environments (Mori et al., 2020; Qin et al., 2019; Tian et al., 2017). Clone libraries of the functional genes *dsrB* and *soxB* were constructed for the SRB and SOB populations, respectively. Quantitative polymerase chain reaction (qPCR) was used to determine the abundance of the SRB and SOB populations in the experiments.

## EXPERIMENTAL PROCEDURES

### Reservoir information

**FIGURE 1:**
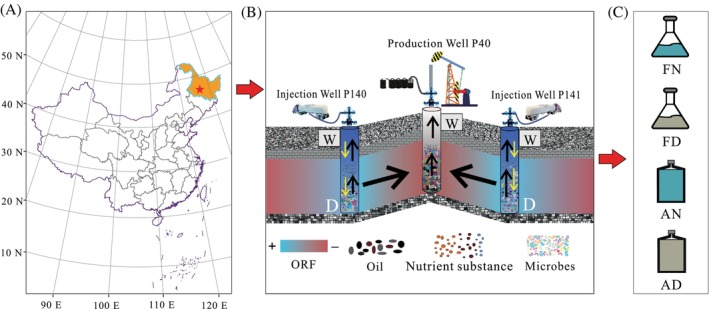
The location of the sampling blocks (A), sampling method (B) and schematic depiction of the experimental design (C). FN: facultative anaerobic conditions with NaNO_3_ (0.6%), FD: facultative anaerobic conditions lack of NaNO_3_, AN: anaerobic conditions with NaNO_3_ (0.6%), AD: facultative anaerobic conditions lack of NaNO_3_.

Samples (oil–water mixture) were collected from the wellhead and downhole of two injection wells (P140 and P141) and one production well (P40) in the N_2_‐field block reservoir area of the Daqing oil field in China. The Daqing oilfield is located in the central part of the Songliao Plain, Heilongjiang Province. It is a large‐scale anticline structural reservoir, and its oil layers are composed of Mesozoic continental Cretaceous sandstone at depths of 900–1200 m. The Daqing petroleum reservoir is characterised by a high wax content, high freezing point, high viscosity and low sulphur content. The average temperature of the Daqing petroleum reservoir is 45°C, with an average permeability of 132 × 10^−3^ μm^2^. The density of the crude oil is 0.846 g cm^−3^, with an oil viscosity of 18 mPa s. The selected injection and production wells are in the same injection–production system where water is injected from the wellhead to the downhole of injection wells P140 and P141, and then flooded to the downhole of production well P40 (Figure 1).

### Sampling and genome extraction

Production mixed oil/water samples were collected directly from each wellhead of P140, P141 and P40 in July 2015, March 2016 and December 2016, while samples were collected from the zone near the downhole of the production wells P140 and P141 in July 2015 and March 2016 by field personnel from PetroChina. The samples from the downhole of the injection wells were obtained by water backflow. A total of 13 samples were collected. To avoid contamination and oxygen intrusion, the samples were used to fill 10 L sterilised plastic bottles, which were immediately sealed with screw caps. The bottles were then transported to the laboratory as soon as possible for further analysis. After the oil and water phases were separated, 5 L of each water sample was centrifuged at 4°C for 15 min at 10,000 × *g* in a high‐speed centrifuge (Beckman, Pasadena, CA) to collect microbial cells, and 100 mL of water was used for chemical analysis. Total genomic DNA was extracted from the cell deposits using methods previously described (Gao et al., 2015).

### Sequencing of partial 16S rRNA genes and potential species analysis

The bacterial 16S rRNA gene V4 region was amplified using the primer sets 515f (5′‐GTG CCA GCM GCC GCG GTA A‐3′) and 907r (5′‐CCG TCA ATT CMT TTR AGT TT‐3′). PCR amplicons were sequenced on an Illumina HiSeq2500 platform at Majorbio BioPharm Technology Co., Ltd., Shanghai, China. Pairs of reads from the original DNA fragments were merged using fast length adjustment of short reads (Magoč & Salzberg, 2011). The raw sequences were demultiplexed and quality‐filtered using the default parameters in the Quantitative Insights Into Microbial Ecology software package to obtain high‐quality clean tags (Bokulich et al., 2013; Caporaso et al., 2010). Chimeric sequences were detected by the UCHIME algorithm and were removed (Edgar et al., 2011). Then, the sequences were classified into operational taxonomic units (OTUs) at 97% similarity using the Uparse pipeline, and representative sequences for each OTU were screened (Edgar, 2013). The resulting representative sequence set was aligned and assigned a taxonomic classification using Ribosomal Database Project (RDP) (Cole et al., 2014). Potential SRB and SOB were selected based on the general species classification.

### Batch incubation

To explore the effect of dissolved oxygen and nitrate on the distribution of SRB and SOB communities in water samples, batch cultures were carried out using water samples collected from three different sites in an injection–production system in July 2015. Mixed water samples from injection wellhead, injection well downhole and production wellhead were incubated following the designed treatments. Four treatments were established based on different nitrate and dissolved oxygen levels: FN, FD, AN and AD, where F represents facultative anaerobic conditions, A represents anaerobic conditions, N represents the addition of NaNO_3_ (0.6%) and D represents a lack of NaNO_3_. The basal medium contained 0.3% (NH_4_)_2_HPO_4_, 0.5% corn steep powder and 2% crude oil. For facultative incubation, each 250 mL Erlenmeyer flask contained 100 mL of water sample and was sealed with a rubber stopper, followed by cultivation in a shaker at 120 rpm and 42°C. In contrast to the treatments under facultative aerobic conditions, for anaerobic treatment, 200 mL cultures were injected into 250 mL deoxygenated anaerobic bottles and statically incubated in a constant temperature incubator. Each sample was prepared in triplicate. After cultivation for 30 days, 5 and 10 mL samples were collected from each Erlenmeyer flask and anaerobic bottle, respectively. Samples from the same treatment were mixed, and 15 samples (including 3 original samples) were obtained. After centrifugation, the cell deposits were used to extract DNA, and the supernatants were collected for chemical analysis.

### Clone library construction for the *dsrB* and *soxB* genes

To analyse the composition of the SRB and SOB communities in water samples treated under four different conditions, *dsrB* and *soxB* gene clone libraries were constructed. The *dsrB* and *soxB* genes were amplified using the DNA extracted from the batch cultures. The primers and protocols used are summarised in Table S1. Thirty clone libraries were constructed as previously described (Tian et al., 2017). In brief, the process involved the acquisition of functional genes, ligation of target fragments into the pMD19T vector, transformation of the recombinant plasmid into *Escherichia coli* DH5α competent cells and verification of the clones. The correct clones were selected for sequencing by an automated ABI 3730 DNA sequencer. After sequencing, the nucleotide sequences of the *dsrB* and *soxB* genes were processed to remove primer and vector sequences using VecScreen, and chimaeras were excluded from the Chimaera Check programme. Valid sequences with a similarity of 85% were assigned to an OTU and the representative sequences of each OTU were selected using Mothur software (Schloss et al., 2009). The representative DNA sequences were subjected to BLAST searches against GenBank to identify their phylogenetic affiliations. Poor‐quality and nonspecific sequences were also excluded from the analysis. Phylogenetic trees based on the *dsrB* and *soxB* genes were constructed by the neighbour‐joining method with 1000 bootstrap replicates using MEGA7 (Kumar et al., 2016) and were visualised via the Interactive Tree of Life v5 web server (Letunic & Bork, 2021).

### Quantitative PCR

To calculate the abundance of SRB, SOB and total bacteria, real‐time fluorescent qPCR was performed on the DNA extracted from the water samples under the four different treatment conditions using the *dsrB*, *soxB* and 16S rRNA genes as molecular markers. The reactions were performed using the Bester SybrGreen qPCR Mastermix (DBI Bioscience, Germany) in a Bio‐Rad iQ5 Sequence Detection System (Applied Biosystems, Carlsbad, CA). The primers and protocols used are summarised in Table S1. Standard curves were constructed by using 10‐fold serial dilutions of standard plasmids with a known number of copies of template DNA. Efficiencies of 90%–110% and corresponding R values >0.99 were considered credible, and gene copy numbers in unknown samples were determined based on standard curves. Samples were prepared in triplicate containing no template controls. The specificity of the PCR amplification was determined by melting curve annealing from 55 to 98°C at the end of each reaction.

### Ion concentration detection

All cell‐free water samples were obtained from the same well and samples were mixed for ion analysis. The concentrations of anions, including SO_4_^2−^, NO_3_^−^, NO_2_^−^ and Ac^−^, in the injection and production waters were analysed using an ion chromatograph (DIONEX ICS‐1000) with a Shim‐pack IC‐C3 column. For batch culture samples, ion concentrations were determined before and 30 days after culture; the utilisation rates of SO_4_^2−^ and NO_3_^−^ were calculated for different treatments.

### Statistical analysis

The alpha diversities of the SRB and SOB communities at the genus/OTU level were calculated, including the number of genera and the Shannon, Simpson, evenness and Chao1 indices. The similarities and differences among the SRB and SOB microbial communities were illustrated by principal coordinate analysis (PCoA) and permutational multivariate analysis of variance (PERMANOVA) based on Bray–Curtis distances using the *vegan* and *ggplot2* packages in R software (version 4.1.2). Redundancy analysis (RDA) and Spearman rank‐order correlation were also conducted to determine correlations between ion concentration and microbial community composition using the *vegan* and *Hmisc* packages in R software (version 4.1.2). Difference analysis of SRB and SOB among multiple samples was performed using statistical analysis of metagenomic profiles (STAMP) software (Parks et al., 2014). Two‐way analysis of variance (ANOVA) was conducted to assess the influence of nitrate, oxygen and their interaction on the abundance of functional genes, the ratio of *dsrB* or *soxB* to the 16S rRNA gene and the ratio of *dsrB* to *soxB* (Tukey's *t*‐test). The inhibitory rate of SRB was assessed by the ratio of *dsrB*/16S rDNA in the treated samples to that in the original samples.

### GenBank submission and accession numbers

All validated nucleotide sequence data obtained from the clone libraries in this study were deposited in the GenBank database under accession numbers ON470224‐ON470303 and ON470304‐ON470359 for the *dsrB* and *soxB* genes, respectively. The raw reads obtained by Illumina MiSeq sequencing were deposited in the Sequence Read Archive at the National Center for Biotechnology Information (BioProject ID: PRJNA 489604).

## RESULTS

### Geochemical characteristics of the water samples

In total, 13 oil/water mixture samples were collected from two injection wells (P140 and P141) and one production well (P40) located in the N_2_ block of the Daqing Reservoir in 2015 and 2016. The distances between P140, P141 and P40 were approximately tens of kilometres, and the locations had different characteristics. The permeabilities of P140, P141 and P40 were 0.244, 0.435 and 0.342 μm^2^, respectively. With water flooding and nutrient injection, the ion concentrations of the water samples changed, as summarised in Table 1. Obvious differences between the samples were observed in the concentration of SO_4_^2−^, which ranged from 8.7 to 48.8 mg/L and could favour the sulphate reduction reaction. Additionally, samples from the downhole of injection wells contained higher levels of SO_4_^2−^ than those from the wellheads of injection and production wells. The concentration of NO_3_^−^ varied from 1.5 to 16.7 mg/L, which was generally lower than the concentration of SO_4_^2−^. Differences were observed in the concentration of NO_2_^−^ among the samples, which ranged from 4.9 to 53.2 mg/L. For the concentration of Ac^−^, no significant difference was detected among the samples, with values fluctuating approximately 10.0 mg/L.

**TABLE 1:** Ion concentrations of water samples collected from P140, P141 and P40 in three different periods.

Ion (mg/L)	Sample												
	July 2015					March 2016					December 2016		
	Wellhead of P140	Downhole of P140	Wellhead of P141	Downhole of P141	Wellhead of P40	Wellhead of P140	Downhole of P140	Wellhead of P141	Downhole of P141	Wellhead of P40	Wellhead of P140	Wellhead of P141	Wellhead of P40
SO_4_^2−^	8.7	45.5	15.4	48.8	11.9	14.5	16.9	18.3	21.1	15.4	9.2	13.7	11.4
NO_3_^−^	2.8	2.2	16.7	1.5	4.4	3.5	12.3	2.3	1.5	5.7	4.8	8.3	7.8
NO_2_^−^	53.2	5.5	8.4	6.7	37.9	43.3	7.6	8.7	4.9	44.7	28.0	18.3	42.2
Ac^−^	10.5	8.9	10.9	9.5	9.8	11.7	10.6	7.3	8.0	10.9	11.6	9.9	10.7

*Note*: P140, P141: two injection wells, and P40: one production well.

### SRB and SOB communities of water samples from reservoirs

**FIGURE 2:**
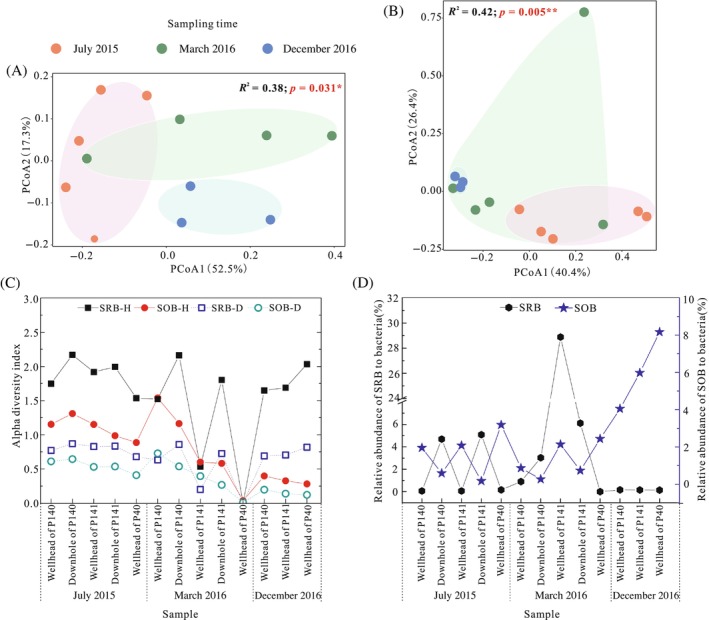
Principal coordinate analysis (PCoA) and PERMANOVA of sulphate‐reducing bacteria (SRB) (A) and sulphur‐oxidising bacteria (SOB) (B) communities inferred from 16S rRNA gene Miseq‐sequencing based on Bray–Curtis distance at the genus level; (C) Shannon (‐H) and Simpson (‐D) indices of SRB and SOB communities inferred from 16S rRNA gene Miseq‐sequencing at the genus level; (D): Relative abundance of potential SRB and SOB to total bacteria calculated by the ratio of potential SRB/SOB reads to total reads.

In total, 23 genera of potential SRB were inferred from the total bacterial community analysis, but no potential SRB were detected in the samples collected from approximately the wellhead of production well P40 in March 2016 (Table S2). PCoA analysis revealed that the SRB community structures were different among the samples collected in the different periods (Figure 2A; Table S3). Among detected potential SRB, *Desulfatirhabdium*, *Desulfobacca*, *Desulfobulbus* and *Desulfovibrio* genera were shared among the samples, accounting for at least 50.0% percentage of the total number of SRB (Figure S1a). For samples collected in July 2015, the average relative abundances of the genera *Desulfobacca*, *Desulfobulbus* and *Desulfovibrio* were relatively high, while for samples collected in March. 2016 and December 2016, the average relative abundance of *Desulfovibrio* increased sharply (by 27.7% and 32.1%) while that of *Desulfobulbus* decreased dramatically (by 13.7% and 24.0%). Analysis of the differences in genera showed that *Desulfurivibrio*, Desulfovibrionaceae_unclassified and Desulfobacteraceae_unclassified were significantly different among samples collected in July 2015, March 2016 and December 2016 (Figure S1c). Additionally, four and five unique genera were found in the samples collected in July 2015 and in the samples collected in March 2016, accounting for up to 17.9% and 1.8%, respectively. However, there was no difference between the injection well and production well samples in terms of the SRB distribution or dominant species at the genus level. Among the single‐well samples, community diversity and evenness in the injection wells were greater for the downhole samples than for the wellhead samples (Figure 2C). As shown in Figure 2D, the relative abundance of total SRB to bacteria was calculated. We found that the samples collected in March 2016 contained a greater relative abundance of SRB than samples collected in July 2015 and March 2016. At the sampling sites, there was a lower proportion of wellhead samples than downhole samples, except for the wellhead sample from the injection well P141 collected in March 2016, which had the highest proportion reaching 28.9%.

**FIGURE 3:**
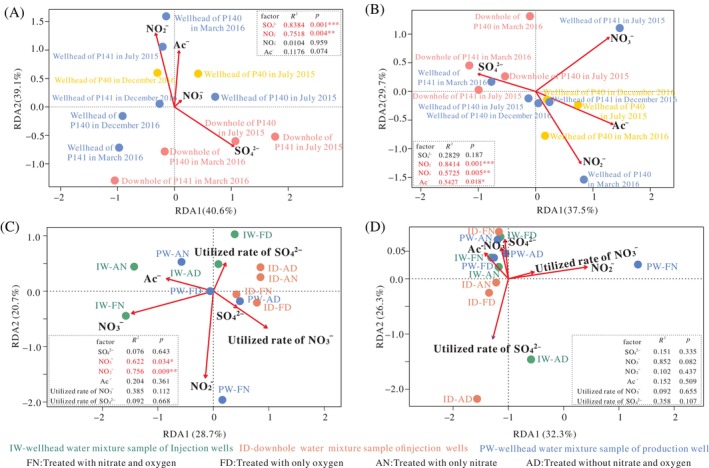
Redundancy analysis (RDA) of the relationships of sample variables with the sulphur‐related microbial communities structure at the genus level. (A) Sulphate‐reducing bacteria (SRB) communities retrieved from 16S rRNA gene Miseq‐sequencing; (B) sulphur‐oxidising bacteria (SOB) communities retrieved from 16S rRNA gene Miseq‐sequencing; (C) SRB communities based on *dsrB* gene clone libraries; (D) SOB communities based on *soxB* gene clone libraries. The sample variables include SO_4_^2−^: concentration of SO_4_^2−^, NO_3_^−^: concentration of NO_3_^−^, NO_2_^−^: concentration of NO_2_^−^, Ac^−^: concentration of Ac^−^, utilised rate of SO_4_^2−^ and utilised rate of NO_3_^−^. *significant at *p* < 0.05, **significant at *p* < 0.01, ***significant at *p* < 0.001.

For potential SOB, 18 genera were obtained. The community assemblies were relatively uneven and shifted significantly among different samples, which was particularly evident during the different sampling periods (Figure 3B). The genera affiliated with the SOB were more closely distributed in the samples collected in July 2015 than in the samples collected in March 2016 and December 2016. In contrast to those of the SRB, no SOB genera were shared among the samples. The abundances of *Sulfuricurvum* and Rhodobacteraceae_unclassified were different among the samples (Figure S1d). For the samples collected in July 2015, *Dechloromonas*, Rhodobacteraceae_unclassified and Ectothiorhodospiraceae_norank were shared accounting for relatively high proportions, ranging from 43.2% to 95.6% (Figure S1b). Additionally, the shared genera were more abundant in the downhole samples than in the wellhead samples. For the samples collected in March 2016, *Dechloromonas* and *Sulfuricurvum* were the dominant genera, while the abundance of Rhodobacteraceae_unclassified decreased, except in the wellhead sample from the production well P40, which almost completely consisted of *Azospirillum*. The SOB communities for the samples collected in December 2016 had similar structures, with *Sulfuricurvum* and *Dechloromonas* accounting for the majority (higher than 95.0%). Additionally, the samples collected in July 2015 and March 2016 contained two and three unique genera, respectively. Like the SRB, no difference was observed between the injection well and production well samples in terms of the SOB communities and dominant species at the genus level. For the *α* diversity indices of potential SOB, the diversity and evenness of the communities in the samples collected in December 2016 were lower than those in the samples collected in July 2015 and March 2016 (Figure 2C). For the relative abundance of total SOB to bacteria, in contrast to the results for SRB, the proportions were greater in the wellhead samples than in the downhole samples (Figure 2D). In addition, the relative abundance of total SOB in the samples collected in December 2016 was significantly greater than that in the samples collected in July 2015 and March 2016.

### Relationships between ion concentrations and the SRB and SOB communities

As shown in Figure 3A, the RDA suggested that there were significant positive correlations between the potential SRB communities and the concentrations of SO_4_^2−^ (*p* = 0.001) and NO_2_^−^ (*p* = 0.004). However, the concentrations of NO_3_^−^ and Ac^−^ did not exhibit a significant correlation with the potential SRB communities (*p* > 0.05). Additionally, the concentrations of SO_4_^2−^ were positively related to the concentrations in the downhole samples, while most of the wellhead samples were affected by the concentrations of NO_2_^−^ and Ac^−^. The potential SOB communities were significantly influenced by the concentrations of NO_2_^−^ (*p* = 0.001), NO_3_^−^ (*p* = 0.005) and Ac^−^ (*p* = 0.018) (Figure 3B). No significant correlation was found between sulphate concentration and the potential SOB communities (*p* > 0.05). Similarly, the community structures of the downhole samples were more strongly affected by the concentration of SO_4_^2−^, while the community structures of the wellhead samples were positively correlated with the concentrations of the other three anions. The RDA for the ion concentrations explained 79.7% and 67.2% of the data variability in the first two axes for SRB and SOB, respectively.

### Responses of the SRB and SOB communities to nitrate and oxygen

For the 15 *dsrB* clone libraries, 704 sequences were ultimately obtained and divided into 80 OTUs, while 716 *soxB* sequences from the 15 clone libraries were assigned to 56 OTUs with a similarity of 85%. For effective *dsrB* sequences, 45, 47 and 23 OTUs were obtained in the water mixture groups from the injection wellhead, injection well downhole, and production wellhead, respectively; for *soxB* sequences, 31, 27 and 14 OTUs were assigned to the water mixture groups of the injection wellhead, injection well downhole and production wellhead, respectively (Table S2). The alpha diversity indices were calculated at both the OTU and genus levels for SRB (Figure S2a–d) and SOB (Figure S2e–h). Nitrate and oxygen had different influences on the diversity indices. For example, the number of observed species and Shannon and Simpson indices of SRB communities increased (Figure S2a–c), while the number of SOB communities decreased in the wellhead water mixture sample from the injection wells (Figure S2a–c). Additionally, samples from different sites exhibited significant variations in their response to the various treatment conditions. The response of the borehole and downhole samples to nitrate and oxygen differed considerably for SRB, whereas for SOB, the response varied significantly between the injection wells and production wells. The phylogenetic trees of the *dsrB* and *soxB* genes, which included seven and nine defined families are shown in Figures S4 and S5.

**FIGURE 4:**
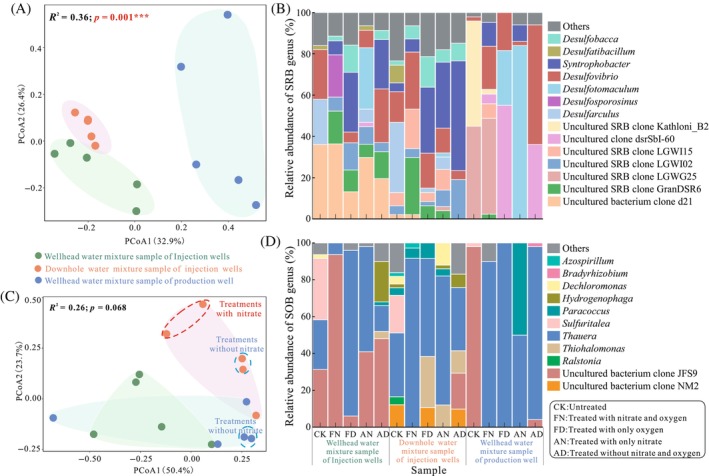
Principal coordinate analysis (PCoA) and PERMANOVA based on Bray–Curtis distance and community structures of sulphate‐reducing bacteria (SRB) (A, B) and sulphur‐oxidising bacteria (SOB) (C, D) communities obtained by *dsrB* and *soxB* genes clone libraries at the genus level.

As illustrated in Figure 4A, PCoA revealed that the SRB communities were closely clustered based on their sampling sites at the genus level (*p* = 0.001). Little variation was observed between the different treatments (*p* > 0.05). For the injection wellhead water mixture group, the uncultured bacterium clone d21, *Desulfovobrio* and *Desulfarculus* made up the majority of the bacteria (Figure 4B). For the treated samples, the percentages of uncultured bacterium clone d21 and *Desulfarculus* decreased to different degrees, while *Syntrophobacter*, uncultured sulphate‐reducing bacterium clone GranDSR6 and clone LGWI02 were present at relatively high percentages. *Desulfarculus* was the dominant genus in the downhole water mixture group of injection wells followed by *Desulfovobrio*. For the treated samples, the FN sample was dominated by *Desulfovobrio*, the uncultured sulphate‐reducing bacterium clone GranDSR6 and the clone LGWI15, while the FD, AN and AD samples were characterised by a high percentage of *Syntrophobacter*, ranging from 31.9% to 53.2%. Considering the community structure of the production wellhead water mixture group, the uncultured sulphate‐reducing bacterium clone LGWG25 (44.9%) and clone Kathloni_B2 (51.0%) made up the majority of the community. After incubation under the four conditions, the structure changed in different ways; the most obvious difference was in the dominant genera. Through difference analysis, we found that four genera were significantly different among the water mixture groups from the wellhead location of injection wells, downhole location of injection wells, and wellhead location of a production well; these included *Desulfobacca*, uncultured bacterium clone d21, clone dsrBF1H07 and uncultured sulphate‐reducing bacterium clone ShibaoSN68 (Figure S3a). Nitrate and oxygen inhibited the relative abundance of *Desulfarculus* (Figure S3b,c).

In contrast to those of the SRB, the SOB community structures of the three sample groups exhibited lower correlations with both the sampling sites and treatments (*p* > 0.05) (Figure 4C). However, for the downhole samples from injection wells and production wellhead water mixture groups, samples were closely clustered within the same nitrate treatment, for example, the samples treated with no nitrate and the samples treated with nitrate. In the injection wellhead water mixture group, *Sulfuritalea* (33.3%), the uncultured bacterium clone JSF9 (31.3%) and *Thauera* (27.1%) were present in high proportions. After incubation, *Sulfuritalea* disappeared in all the samples, while the uncultured bacterium clone JSF9 was enriched in the FN sample (93.8%), AN sample (40.8%) and AD sample (48.0%). *Thauera* increased in both the FD sample (90.0%) and the AN sample (57.1%). For the injection well downhole water mixture group, *Thauera* constituted the majority, followed by *Sulfuritalea*. Like in the injection wellhead water mixture group, the majority of the community in the treated samples consisted of *Thauera*, and their biodiversity decreased. After incubation, *Thiohalomonas* was detected in the treated samples, accounting for approximately 12.2%–27.7% of the total bacteria, except in the FN sample. For the production wellhead water mixture group, the uncultured bacterium clone JSF9 was the predominated bacterium (98.0%), the abundance of which decreased after incubation, while *Thauera*, which was strongly enriched up to 50.0%–100.0%, was absent in the untreated sample. In the AN sample, *Paracoccus* was present at 50.0%. The statistical analysis revealed that the abundance of the uncultured bacterium clone NM2 significantly differed among the three sample groups (*p* = 0.017), while the abundances of *Sulfuritalea*, the uncultured bacterium clone JFS16 and the clone BROK differed markedly from those in the original sample after treatment with nitrate and oxygen (Figure S3d–f).

### Correlations between sample properties, utilisation rates and community distribution

**FIGURE 5:**
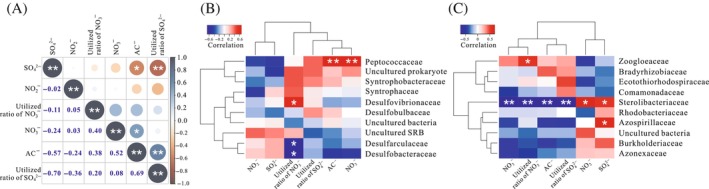
Spearman correlation coefficients (*r*) between the concentrations of ions and utilisation rates of SO_4_^2−^ or NO_3_^−^ (A); between the sample properties and SRB communities (B) or SOB communities (C) at the family level, *significant at *p* < 0.05, **significant at *p* < 0.01.

After incubation, the concentrations of SO_4_^2−^, NO_3_^−^, NO_2_^−^ and Ac^−^ coupled with the utilisation rates of NO_3_^−^ and SO_4_^2−^ were determined for the treated samples (Table 2). The concentrations of SO_4_^2−^ and NO_3_^−^ ranged from 0.0 to 37.3 ± 1.0 mg/L and 27.6 ± 0.9 to 226.1 ± 8.1 mg/L, respectively. The utilisation rates of SO_4_^2−^ and NO_3_^−^ differed between the samples treated with nitrate (‐N) and samples without nitrate (‐D), varying from 19.2 ± 0.5% to 100.0% and 2.6 ± 0.1% to 96.5 ± 3.2%, respectively. NO_2_^−^ was not detected in most of the samples, except for samples treated with nitrate in the water mixture groups from the wellheads of the injection wells and production wells. The concentrations of Ac^−^ fluctuated between 713.5 ± 42.8 and 1713.5 ± 94.2 mg/L. In terms of the relationships between sample properties, the utilisation rate of SO_4_^2−^ was positively correlated with the concentration of Ac^−^ (*r* = 0.69, *p* < 0.05) but negatively correlated with the concentration of SO_4_^2−^ (*r* = −0.70, *p* < 0.01) (Figure 5A). The levels of SO_4_^2−^ and NO_3_^−^ had some effect on the utilisation rate of NO_3_^−^, but there was no obvious correlation (*p* > 0.05). The concentration of Ac^−^ was positively correlated with that of NO_3_^−^ (*r* = 0.52, *p* < 0.05), but negatively correlated with that of SO_4_^2−^ (*r* = −0.57, *p* < 0.05).

**TABLE 2:** Ion concentrations and utilisation rates of nitrate and sulphate for samples under different treatments.

Sample		Ion					
		SO_4_^2−^ (mg/L)	NO_3_^−^ (mg/L)	NO_2_^−^ (mg/L)	AC^−^ (mg/L)	Utilisation rate of SO_4_^2−^ (%)	Utilisation rate of NO_3_^−^ (%)
Injection wellhead water mixture group	FN	9.2 ± 0.3	226.1 ± 8.1	21.1 ± 0.9	1395.8 ± 72.6	23.8 ± 0.9	81.0 ± 2.9
	AN	7.9 ± 0.4	55.3 ± 2.3	nd	1072.9 ± 45.1	34.5 ± 1.6	95.3 ± 4.0
	FD	0.0	95.5 ± 4.1	nd	1302.1 ± 40.4	100.0	2.6 ± 0.1
	AD	0.0	36.4 ± 1.4	nd	776.0 ± 27.2	100.0	62.8 ± 2.4
Injection well downhole water mixture group	FN	37.3 ± 1.0	37.7 ± 1.7	nd	750.0 ± 27.8	19.2 ± 0.5	94.5 ± 4.3
	AN	17.8 ± 0.5	77.9 ± 2.3	nd	765.6 ± 32.9	61.4 ± 1.8	88.7 ± 2.6
	FD	0.0	41.5 ± 2.0	nd	1151.0 ± 55.2	100.0	54.4 ± 2.6
	AD	0.0	27.6 ± 0.9	nd	1036.5 ± 54.9	100.0	67.9 ± 2.2
Production wellhead water mixture group	FN	6.4 ± 0.3	67.8 ± 3.1	103.5 ± 5.3	901.0 ± 44.1	46.0 ± 2.4	96.5 ± 3.2
	AN	4.5 ± 0.2	95.5 ± 4.9	nd	713.5 ± 42.8	61.9 ± 3.0	95.1 ± 3.7
	FD	3.8 ± 0.2	62.8 ± 4.1	nd	1468.8 ± 60.2	68.3 ± 4.1	26.5 ± 1.7
	AD	0.0	38.9 ± 2.3	nd	1713.5 ± 94.2	100.0	54.4 ± 3.2

Abbreviation: nd, not detected.

Based on the RDA, we found that the concentrations of NO_3_^−^ (*p* < 0.05) and NO_2_^−^ (*p* < 0.01) had dramatic effects on the SRB communities at the genus level in the treated samples (Figure 3C). For the SOB communities (Figure 3D), most of the samples were related to the levels of NO_3_^−^, Ac^−^ and SO_4_^2−^; the utilisation rate of SO_4_^2−^ affected the samples containing no nitrate, including the FD and AD samples of the water mixture group of injection well downhole and the AD sample of the water mixture group of injection wellhead. However, the influence of the selected sample properties on the SOB community structure was not significant (*p* > 0.05). For SRB at the family level, Peptococcaceae was positively related to the concentrations of Ac^−^ and NO_3_^−^ (*p* < 0.01); Desulfovibrionaceae, Desulfarculaceae and Desulfobacteraceae were significantly correlated with the utilisation rate of NO_3_^−^ (*p* < 0.05) (Figure 5B). As shown in Figure 5C, the abundance of Sterolibacteriaceae was significantly associated with all the selected factors (*p* < 0.05); the abundances of Zoogloeaceae and Azospirillaceae were positively related to the concentration of SO_4_^2−^ (*p* < 0.05) and the utilisation rate of NO_2_^−^ (*p* < 0.05), respectively.

### Results of quantitative real‐time PCR

**FIGURE 6:**
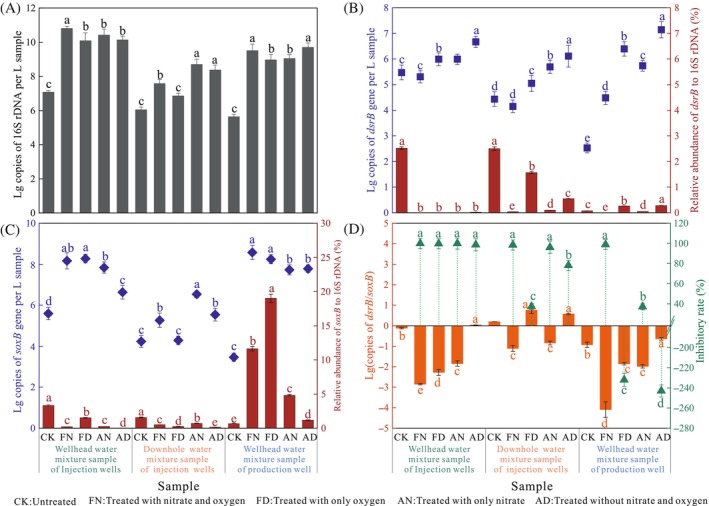
Results of quantitative polymerase chain reaction. (A) Abundance of copy numbers of 16S rRNA gene; (B) abundance of copy numbers of *dsrB* gene and the *dsrB* copies/16S rRNA copies ratio; (C) abundance of copy numbers of *soxB* gene and the *soxB* copies/16S rRNA copies ratio; (D) *dsrB* copies/*soxB* copies ratio and inhibitory rate for sulphate‐reducing bacteria. Error bars represent standard errors of the means (*n* = 3). Different lowercase letters indicate significant differences (*p* < 0.05) among treatments as determined by Tukey's Honest Significant Difference (HSD) method.

After incubation, bacterial growth was stimulated by up to four orders of magnitude compared with the initial samples (Figure 6A). There were differences to some extent among the four treatments in terms of the number of copies of the 16S rRNA gene, and these differences were associated with different conditions in the three groups according to two‐way ANOVA (Table 3). The abundance of *dsrB* increased in all the treated samples except for the FN‐treated samples for the injection wellhead and downhole water mixture groups, while for the production wellhead water mixture group, all the treatments stimulated SRB, which increased in abundance by two to four times. Nitrate and oxygen had notable effects on the number of *dsrB* copies in the three groups (*p* < 0.001). Like that of the *dsrB* gene, the increasing trend was more evident as the abundance of *soxB* was two to five times greater than that in the initial samples and was markedly affected by nitrate and oxygen, except in the production wellhead water mixture group. Here, we determined the ratio of *dsrB* or *soxB* copies to 16S rDNA copies (*dsrB*% or *soxB*%) to measure the relative abundance of SRB or SOB. Obvious differences were observed in the relative abundance of SRB, which dramatically decreased, especially for the samples treated with nitrate and oxygen (Figure 6B). The relative abundances of SOB decreased in response to the presence of nitrate and oxygen in the groups of injection wellhead and downhole water mixtures but increased dramatically in the production wellhead water mixture group, especially for the FD sample (Figure 6C). Nitrate, oxygen and their interaction markedly influenced the relative abundances of SRB and SOB in the three groups (*p* < 0.001). To consider the balance between SOB and SRB abundances, the *dsrB*/*soxB* ratio was calculated and found to vary among the treatments; the lowest value was obtained under the FN treatment for the three groups (Figure 6D). The nitrate content had an obvious influence on the *dsrB*/*soxB* ratio (*p* < 0.001). Additionally, the *dsrB*% of the treated samples was compared with that of the original samples to evaluate the inhibitory rates of the SRB (Figure 6D). The different treatments had inhibitory effects on *dsrB*%, except for the production wellhead water mixture sample treated with no nitrate, for which the maximum inhibition rate reached 99.9 ± 0.1%, 99.7 ± 0.2%, 98.8 ± 0.2% for samples treated with high nitrate and oxygen levels in the wellhead water mixture group of the injection wells, the downhole water mixture group of the injection wells and the wellhead water mixture group of the production well, respectively. In comparison, nitrate and oxygen had different inhibitory effects on the three groups. The same degree of inhibition was achieved at different nitrate and oxygen levels for the wellhead water mixture group of the injection wells, stronger inhibitory effects were observed with the addition of nitrate rather than with oxygen for the wellhead water mixture group of the production well, while oxygen had greater inhibition in samples treated with no nitrate than in those with nitrate in the downhole water mixture group of the injection wells.

**TABLE 3:** Two‐way ANOVA analysis of nitrate and oxygen on the abundance of functional genes.

Group	Functional genes	Nitrate	Oxygen	Interaction
Injection wellhead water mixture group	*dsrB*	***	***	ns
	*soxB*	***	***	***
	16S rDNA	***	ns	*
	*dsrB*/16S rDNA	***	***	***
	*soxB*/16S rDNA	***	***	***
	*dsrB*/*soxB*	***	***	ns
Injection well downhole water mixture group	*dsrB*	***	***	*
	*soxB*	***	**	ns
	16S rDNA	***	***	*
	*dsrB*/16S rDNA	***	***	***
	*soxB*/16S rDNA	***	***	***
	*dsrB*/*soxB*	***	ns	*
Production wellhead water mixture group	*dsrB*	***	***	*
	*soxB*	ns	***	***
	16S rDNA	ns	ns	***
	*dsrB*/16S rDNA	***	***	***
	*soxB*/16S rDNA	***	***	***
	*dsrB*/*soxB*	***	***	***

* *p* < 0.05 at *α* = 0.05 level;

** *p* < 0.01 at *α* = 0.05 level;

*** *p* < 0.001 at *α* = 0.05 level; ns represents no significant relationship at *α* = 0.05 level.

## DISCUSSION

Significant differences were found in the SRB and SOB communities among the sampling periods based on the bacterial community analysis (Figure 2; Table S3), indicating that the influence of the sampling period was greater than that of sampling well stratigraphy. For SRB, *Desulfovibrio* and *Desulfobulbus* were the common dominant species in samples from injection wells and production wells, and these species are commonly detected in reservoir habitats (An et al., 2017; Gao & Fan, 2023; Li et al., 2017; Marietou, 2021; Tian et al., 2017; Zhou et al., 2023). Many studies have shown that members of the *Desulfovibrio* genus use metallic iron as the sole electron donor and accelerate carbon steel corrosion (Chatterjee et al., 2021; Jia et al., 2018; Sharma et al., 2018). Jia et al. (2018) reported that MIC via *Desulfovibrio vulgaris* in anaerobic vials occurred due to the cross‐cell wall electron transfer rather than due to biogenic H_2_S during a 7‐day incubation period. Members of *Desulfobulbus* could accelerate corrosion since these species can grow with Fe(III) (Holmes et al., 2004). For SOB, unclassified bacteria affiliated with *Rhodobacteraceae* constituted most of the communities in the samples collected in June 2015, with their abundance decreasing sharply during flooding, while the abundances of *Dechloromonas* and *Sulfuricurvum* increased markedly and dominated the samples collected in March 2016 and December 2016, respectively. The increase in *Dechloromonas* abundance may have resulted from the ability of these bacteria to degrade hydrocarbons and respire NO_3_^−^ and/or NO_2_^−^ (Coates et al., 2001; Duffner et al., 2021). Ray et al. (2018) reported that *Dechloromonas aromatica* could catalyse the oxidation of thiosulphate, via the interaction of the SoxB and SoxYZ proteins. Species of *Sulfuricurvum* utilising sulphide, elemental sulphur, thiosulphate and hydrogen as electron donors and nitrate as an electron acceptor have been isolated from underground crude oil storage cavities under anaerobic conditions (Kodama & Watanabe, 2004). No unique genera were found in the production well samples, while 12 and 11 genera were detected in the injection well samples for SRB and SOB, respectively. These results indicate that the sulphur cycle‐related microbial communities of injection wells could affect those of production wells to some extent, as injected microorganisms can be transported to production wells due to the water injection pressure, low permeability and short injection–production well spacing. For the relative abundance of SRB and SOB compared to total bacteria, opposite trends were observed, which was obvious in the wellhead and downhole samples. These differences are attributed to the physiological characteristics of the microorganisms, that is, most SRB are strictly anaerobic, while SOB can grow under both aerobic and anaerobic conditions. Previous studies have shown that oxygen strongly influences the composition of microbial populations when injected water flows into production wells (Gao et al., 2015).

After treatment with nitrate and oxygen, the SRB community assembly of the samples changed, especially that of the dominant species (Figure 4A,B). Numerous studies have shown that nitrate changes the SRB composition in petroleum reservoirs (An et al., 2017; Bødtker et al., 2008; Carlson & Hubert, 2019; Marietou et al., 2020; Qi et al., 2022). The relative abundance of *Desulfarculus*, a sulphate‐reducing bacterium that oxidises organic substrates completely to CO_2_, was greater in the nitrate‐treated samples than in the samples with no nitrate (Figure S3c). Oxygen can significantly inhibit bacteria in the genus *Desulfarculus*, which are strictly anaerobic sulphate reducers (Figure S3b) (Sun et al., 2010). A comparison of the species diversity showed that the structures of the SRB communities changed more with the sampling site than with other factors (Figure S2). Additionally, based on our results, there was no obvious species specificity in the inhibitory effects of nitrate and oxygen on SRB (Figure 4A). Different responses to nitrate and oxygen were observed for the SOB communities, whose responses were simplified by the different treatments compared with those of the SRB communities, especially for the concentration of nitrate. Remarkably, most of the sequences detected for the *soxB* gene in the treated samples were affiliated with *Thauera*, the main functional heterotrophic nitrate‐reducing bacteria (hNRB) that inhibits SRB after nitrate injection in the reservoir environment (Bødtker et al., 2008; Hubert & Voordouw, 2007; Suri et al., 2017). Additionally, the uncultured bacterium clone JFS9, which was dominant in the injection wellhead water mixture, is closely related to *Thauera humireducens* SgZ‐1, which can reduce a humus analogue, humic acids, soluble Fe(III) and Fe(III) oxides (Ma et al., 2015). Regardless of the oxygen content, *Thauera* was stimulated by nitrate, as these species are capable of performing denitrification using a variety of carbon sources, including aromatic compounds (Ren et al., 2021; Shinoda et al., 2004). *Thiohalomonas*, an autotrophic denitrifier, can catalyse thiosulphate oxidation and nitrate reduction simultaneously and was found to be highly abundant in treated samples (Shao et al., 2011). *Paracoccus* was strongly enriched in the AN sample from the production wellhead water mixture group, and members of this genus exhibited efficient aerobic denitrification and carbon removal abilities (Chen et al., 2020; Zhang et al., 2020). *Sulfuricurvum*, *Sulfurospirillum* and *Sulfurovum*, which are typical SOB genera, were not stimulated in any of the treatments, and they exhibited obvious differences between the original and treated samples but had very low relative abundances in treatments with nitrate and oxygen.

Inorganic substances can serve as electron donors or acceptors for microorganisms, exerting an influence on microbial communities. Here, we found that the concentration of sulphate could markedly affect the SRB communities of samples collected from oil fields during water flooding but had no significant influence on the SRB communities after incubation. However, the nitrite content affected the SRB communities in all the samples (*p* < 0.01). Previous studies confirmed that nitrite could inhibit the activity of SRB because it inhibits sulphite reduction by competing with the sulphite reductase enzyme and has been used to control SRB‐related biofouling in oil fields (Haveman et al., 2004; Kaster et al., 2007). Moreover, An et al. (2017) and Marietou et al. (2020) confirmed that nitrite inhibition is the most effective nitrate‐based souring mitigation mechanism. The SOB distributions of samples collected from oil fields were affected by the levels of nitrate and nitrite, which could stimulate the growth of NR‐SOB (Greene et al., 2003; Hubert et al., 2005; Jahanbani Veshareh & Nick, 2019; Qi et al., 2022). In addition, the concentration of acetate had an obvious influence on the SOB communities. Wu et al. (2019) reported that acetate could stimulate sulphur cycling and alter the community structure of SRB and SOB as acetate is known to be used by many SRB and SOB. However, the SOB compositions of the treated samples were not significantly affected by the concentrations of the detected ions (*p* > 0.05).

Microbial abundance is another important index for evaluating the activity of microbial communities. Zhou et al. (2023) confirmed that corrosion rates were positively correlated with the abundance of sulphate‐reducing prokaryotes. In comparison to community structure, nitrate and oxygen had more significant influences on the copy numbers and relative abundances of functional genes in the treated samples (*p* < 0.05) (Figure 6; Table 3). In the production wells, the number of copies of the *dsrB* and *soxB* genes increased more than that in the injection wells after treatment. The highest SRB inhibition rate and the lowest *dsrB*/*soxB* ratio were obtained under the FN conditions in the three groups, suggesting that the synergistic effect of high nitrate and high oxygen levels on the inhibition of SRB by potential SOB was strong, which confirmed that nitrate and oxygen are the main factors preventing the growth of SRB and the occurrence of corrosion (An et al., 2017; Hubert & Voordouw, 2007; Okpala & Voordouw, 2018). Furthermore, an abundance analysis of the functional community is needed to better understand how these bacteria respond to stress, especially in terms of relative abundance, which could partly reflect the metabolic intensity of total bacteria. However, interestingly, the dominant genera, including *Thauera*, *Thiohalomonas* and *Paracoccus*, detected in the treated samples based on the *soxB* gene were affiliated with the NRB. Based on the quantitative analysis of the *napA* gene in the samples (Feng et al., 2011), as shown in Figure S6, the trend in the *napA* copy number was similar to that in the *soxB* copy number, especially in water injection wells, and the copy number of *napA* was lower than that of *soxB*. Notably, researchers have found that NRB have the same characteristics as biological cathodes in catalytic reduction, affecting the corrosion of SRB and causing the corrosion of metals (Jia et al., 2017a, 2017b; Yuk et al., 2020). Therefore, are hNRB or NR‐SOB the microbes that determine the inhibition of SRB growth? What is the relationship between hNRBs and NR‐SOB? To answer these questions, the types of functional bacteria that inhibit the growth of SRB and the role of SOB in this process need to be further studied.

## CONCLUSION

Petroleum reservoirs, which are injection–production systems, are faced with serious corrosion and souring issues caused mainly by SRB, and effective corrosion mitigation measures are urgently needed. In this context, coupled nitrate and oxygen injection is a widely used strategy. No significant difference in the SRB and SOB community structures was observed between injection and production well samples in this study, indicating that the sulphur‐related microbial community of injection wells could affect that of production well. Nitrate and oxygen shape sulphur‐related microbial community structures, especially those of SOB communities. The copy numbers, relative abundances of functional genes and inhibitory rates were significantly changed by nitrate and oxygen. Additionally, nitrate and oxygen had different inhibitory effects on each treatment group. Among the four treatments, the synergistic effect of high nitrate and oxygen levels was strong on the inhibition of SRB by potential SOB. These results provide important fundamental insights into the response of sulphur‐related microbial communities to nitrate and oxygen in petroleum reservoirs.

## AUTHOR CONTRIBUTIONS

**Huimei Tian:** Conceptualization (lead); formal analysis (lead); funding acquisition (equal); investigation (equal); writing – original draft (lead). **Peike Gao:** Investigation (equal); methodology (equal); validation (lead); visualization (lead). **Chen Qi:** Investigation (equal); methodology (equal). **Guoqiang Li:** Project administration (lead); writing – review and editing (supporting). **Ting Ma:** Conceptualization (supporting); funding acquisition (equal); resources (lead); supervision (lead); writing – review and editing (lead).

## CONFLICT OF INTEREST STATEMENT

The authors declare no conflicts of interest.

## Supporting information


**Data S1.** Supporting information.

## Data Availability

Illumina amplicon reads generated in this study have been deposited in GenBank under BioProject number PRJNA489604 and the validated nucleotide sequence data obtained from clone libraries were deposited in the GenBank database with the accession numbers ON470224‐ON470303 and ON470304‐ON470359 for the *dsrB* and *soxB* genes, respectively.
