# Supplementary material for: Nitrate and oxygen significantly changed the abundance rather than structure of sulphate‐reducing and sulphur‐oxidising bacteria in water retrieved from petroleum reservoirs
Source: Environ Microbiol Rep. 2024 Apr 5;16(2):e13248. doi: 10.1111/1758-2229.13248 (PMC10997955; doi:10.1111/1758-2229.13248)
Supplement: Supplementary file 1 — Data S1. Supporting information. [file EMI4-16-e13248-s001.docx]

**Table S1** Protocols used to amplify the 16S rRNA, *dsrB,* and *soxB* genes used in this study

| Genes | Application | Primers | Sequence (5′to 3′) | Protocol | Concentration | Target size | Reference |
| --- | --- | --- | --- | --- | --- | --- | --- |
| 16S rRNA | qPCR | 8F | AGAGTTTGATYMTGGCTC | Initial denaturation at 95°C for 3 min followed by 40 cycles at 94°C for 30 s, 55°C for 30 s, and 72°C for 30 s | 0.3 µM | 300 bp | Gittel et al., 2009 |
|  |  | 338R | GCTGCCTCCCGTAGGAGT |  |  |  |  |
| *dsrB* | Clone library and qPCR | DSRp2060F | CAACATCGTYCAYACCCAGGG | Initial denaturation at 95°C for 3 min followed by 30 (40) cycles of 95°C for 45 s, 55°C for 1 min, and 72°C for 1 min | 0.4 µM | 380 bp | Geets et al., 2006; |
|  |  | DSR4R | GTGTAGCAGTTACCGCA |  |  |  | Wagner et al., 1998 |
| *soxB* | Clone library and qPCR | 710F | ATCGGYCAGGCYTTYCCSTA | Initial denaturation at 95°C for 5 min followed by 30 (40) cycles of 95°C for 30 s, 55°C for 30s, and 72°C for 40 s | 0.4 µM | 510 bp | Tourna et al., 2014 |
|  |  | 1184R | MAVGTGCCGTTGAARTTGC |  |  |  |  |

**References:**

Geets, J., Borremans, B., Diels, L., Springael, D., Vangronsveld, J., van der Lelie, D., Vanbroekhoven, K. (2006) DsrB gene-based DGGE for community and diversity surveys of sulfate-reducing bacteria. J Microbiol Methods 66(2), 194–205.

Gittel, A., Sørensen, K. B., Skovhus, T. L., Ingvorsen, K., Schramm, A. (2009) Prokaryotic community structure and sulfate reducer activity in water from high-temperature oil reservoirs with and without nitrate treatment. Appl Environ Microbiol 75(22), 7086–7096.

Tourna, M., Maclean, P., Condron, L., O'Callaghan, M., Wakelin, S.A. (2014) Links between sulphur oxidation and sulphur-oxidising bacteria abundance and diversity in soil microcosms based on *soxB* functional gene analysis. FEMS Microbiol Ecol 88 (3), 538–549

Wagner, M., Roger, A. J., Flax, J. L., Brusseau, G. A., Stahl, D. A. (1998) Phylogeny of dissimilatory sulfite reductases supports an early origin of sulfate respiration. J Bacteriol 180(11), 2975–2982.

**Table S2** Alpha diversity of samples based on the SRB and SOB communities inferred from 16S rRNA genes Miseq-sequencing.

| Sample | | Genera | | Simpson | | Shannon | | Evenness | | Chao1 | |
| --- | --- | --- | --- | --- | --- | --- | --- | --- | --- | --- | --- |
|  |  | SRB | SOB | SRB | SOB | SRB | SOB | SRB | SOB | SRB | SOB |
| Collected in Jun. 2015 | Wellhead of P140 | 8.0 | 7.0 | 0.8 | 0.6 | 1.7 | 1.2 | 0.7 | 0.5 | 8.0 | 7.0 |
|  | Downhole of P140 | 13.0 | 9.0 | 0.9 | 0.6 | 2.2 | 1.3 | 0.7 | 0.4 | 13.0 | 9.0 |
|  | Wellhead of P141 | 8.0 | 9.0 | 0.8 | 0.5 | 1.9 | 1.2 | 0.9 | 0.4 | 8.0 | 9.0 |
|  | Downhole of P141 | 15.0 | 5.0 | 0.8 | 0.5 | 2.0 | 1.0 | 0.5 | 0.5 | 15.0 | 5.0 |
|  | Wellhead of P40 | 8.0 | 11.0 | 0.7 | 0.4 | 1.5 | 0.9 | 0.6 | 0.2 | 8.0 | 11.0 |
| Collected in Mar. 2016 | Wellhead of P140 | 14.0 | 9.0 | 0.6 | 0.7 | 1.5 | 1.5 | 0.3 | 0.5 | 14.0 | 9.0 |
|  | Downhole of P140 | 13.0 | 7.0 | 0.9 | 0.5 | 2.2 | 1.2 | 0.7 | 0.5 | 13.0 | 7.0 |
|  | Wellhead of P141 | 17.0 | 3.0 | 0.2 | 0.4 | 0.5 | 0.6 | 0.1 | 0.6 | 17.0 | 3.0 |
|  | Downhole of P141 | 15.0 | 6.0 | 0.7 | 0.3 | 1.8 | 0.6 | 0.4 | 0.3 | 15.0 | 6.0 |
|  | Wellhead of P40 | nd | 3.0 | nd | 0.0 | nd | 0.0 | nd | 0.3 | nd | 3.0 |
| Collected in Dec. 2016 | Wellhead of P140 | 10.0 | 6.0 | 0.7 | 0.2 | 1.6 | 0.4 | 0.5 | 0.2 | 10.0 | 6.0 |
|  | Wellhead of P141 | 10.0 | 7.0 | 0.7 | 0.1 | 1.7 | 0.3 | 0.5 | 0.2 | 10.0 | 7.0 |
|  | Wellhead of P40 | 11.0 | 6.0 | 0.8 | 0.1 | 2.0 | 0.3 | 0.7 | 0.2 | 11.0 | 6.0 |

P140, P141 and P40: three production wells. nd means no genus affiliated with SRB was detected.

**Table S3** Pairwise comparison between samples collected in Jul. 2015, Mar. 2016 and Dec. 2016 for the SRB and SOB communities.

|  | SRB community composition | | | SOB community composition | | |
| --- | --- | --- | --- | --- | --- | --- |
| pairs | R^2^ | *p*.value | *p*.adjusted^a^ | R^2^ | *p*.value | *p*.adjusted^a^ |
| samples collected in Jul. 2015 vs samples collected in Mar. 2016 | 0.283 | 0.064 | 0.096 | 0.177 | 0.156 | 0.156 |
| samples collected in Jul. 2015 vs samples collected in Dec. 2016 | 0.546 | 0.022 | 0.066 | 0.546 | 0.028 | 0.042 |
| samples collected in Mar. 2016 vs samples collected in Dec. 2016 | 0.126 | 0.700 | 0.700 | 0.405 | 0.019 | 0.042 |

^a^ *p* values were adjusted by BH.


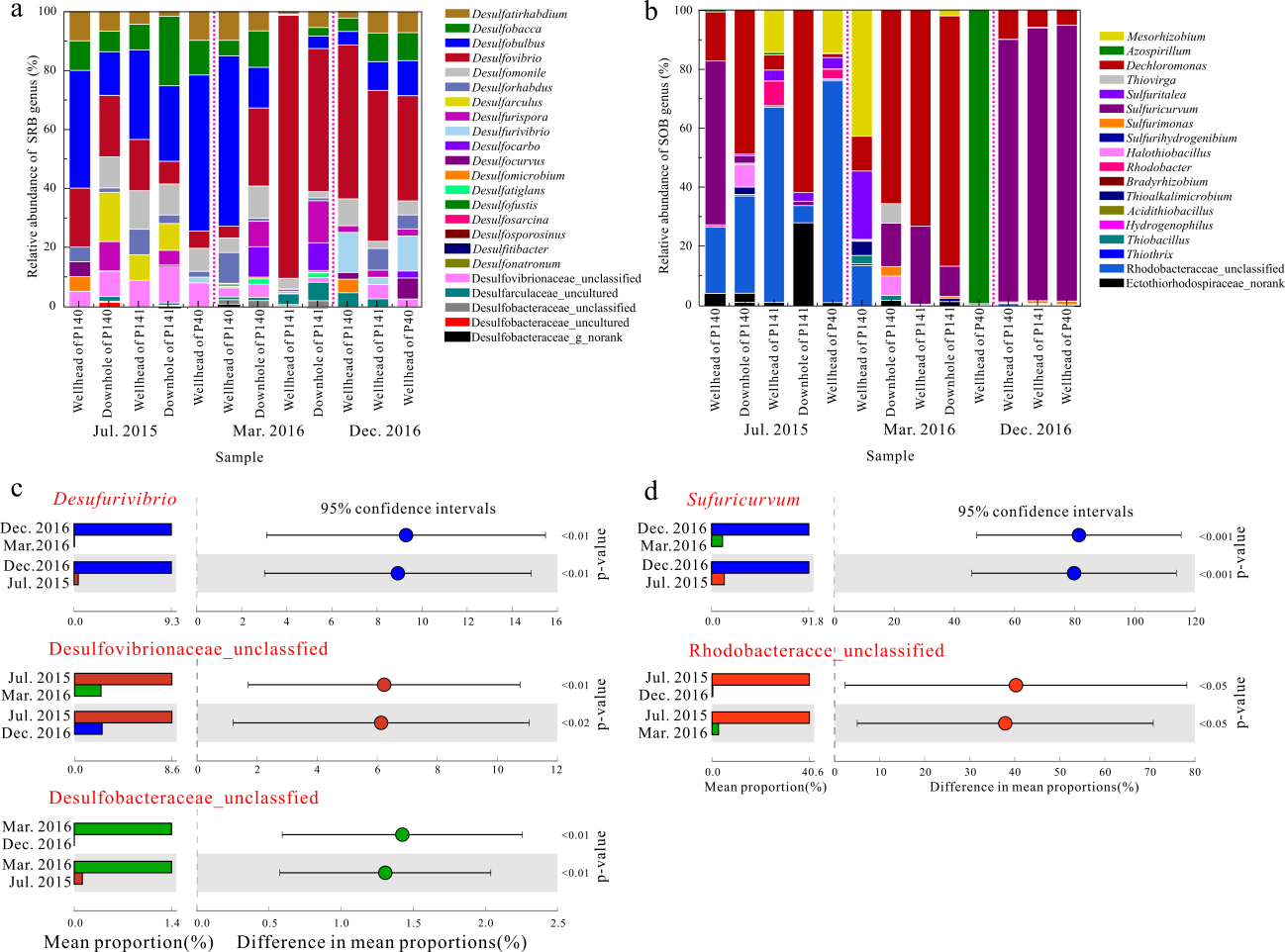


Fig. S1 Relative abundance of sulfur-related bacterial communities of samples at genus level inferred from 16S rRNA Genes Miseq-sequencing (a-SRB, b-SOB) and difference genera among sampling periods at 95% confidence intervals (a-SRB, b-SOB). Samples collected in Jul. 2015, Mar. 2016 and Dec. 2016 were marked as a, b and c, respectively.


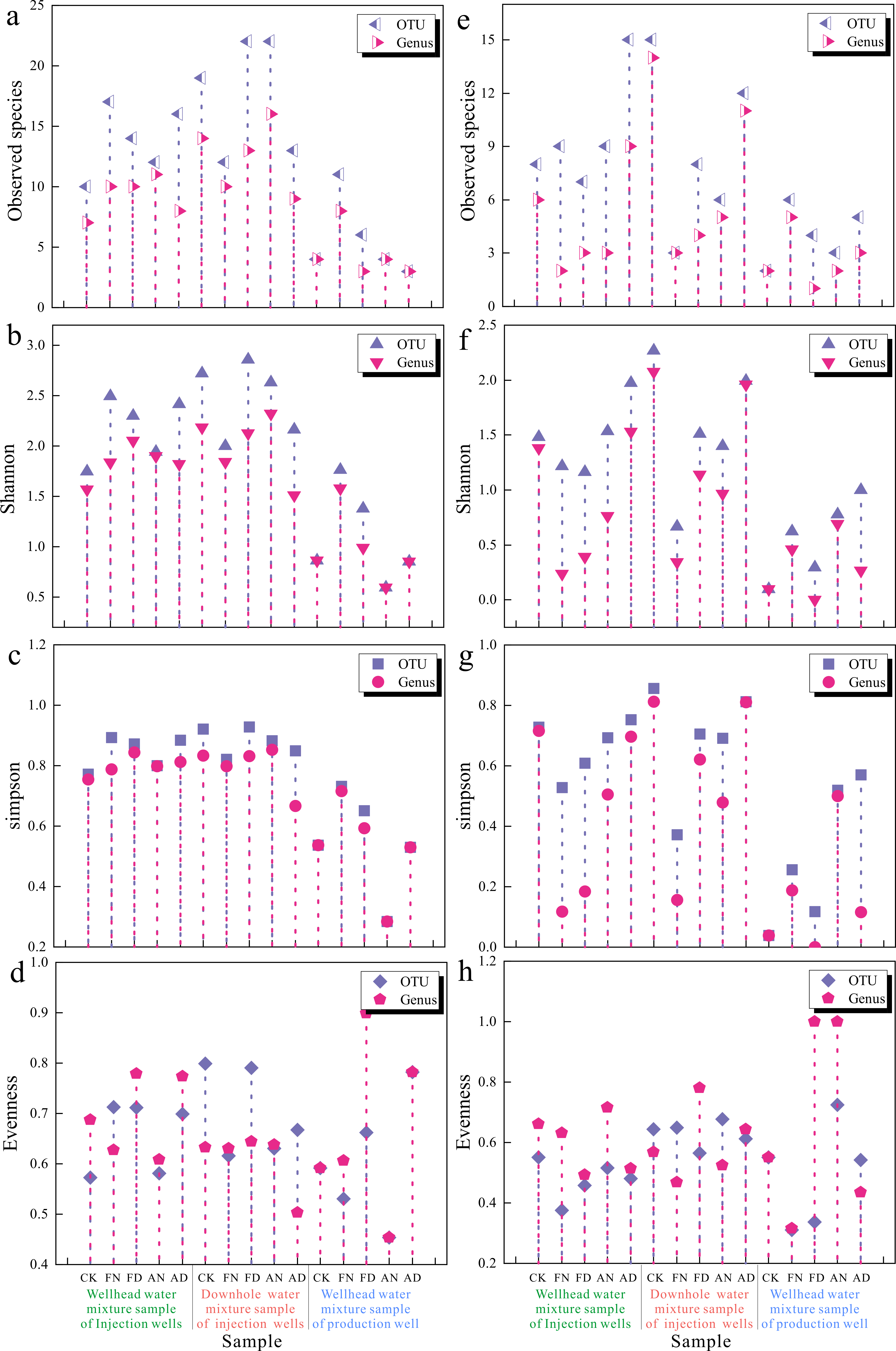


Fig. S2 Alpha diversity indices of samples under different treatments at OTU and genus level. a and e, Observed species; b and f, Shannon; c and g, Simpson; d and h, Evenness, a-d for SRB, e-h for SOB.


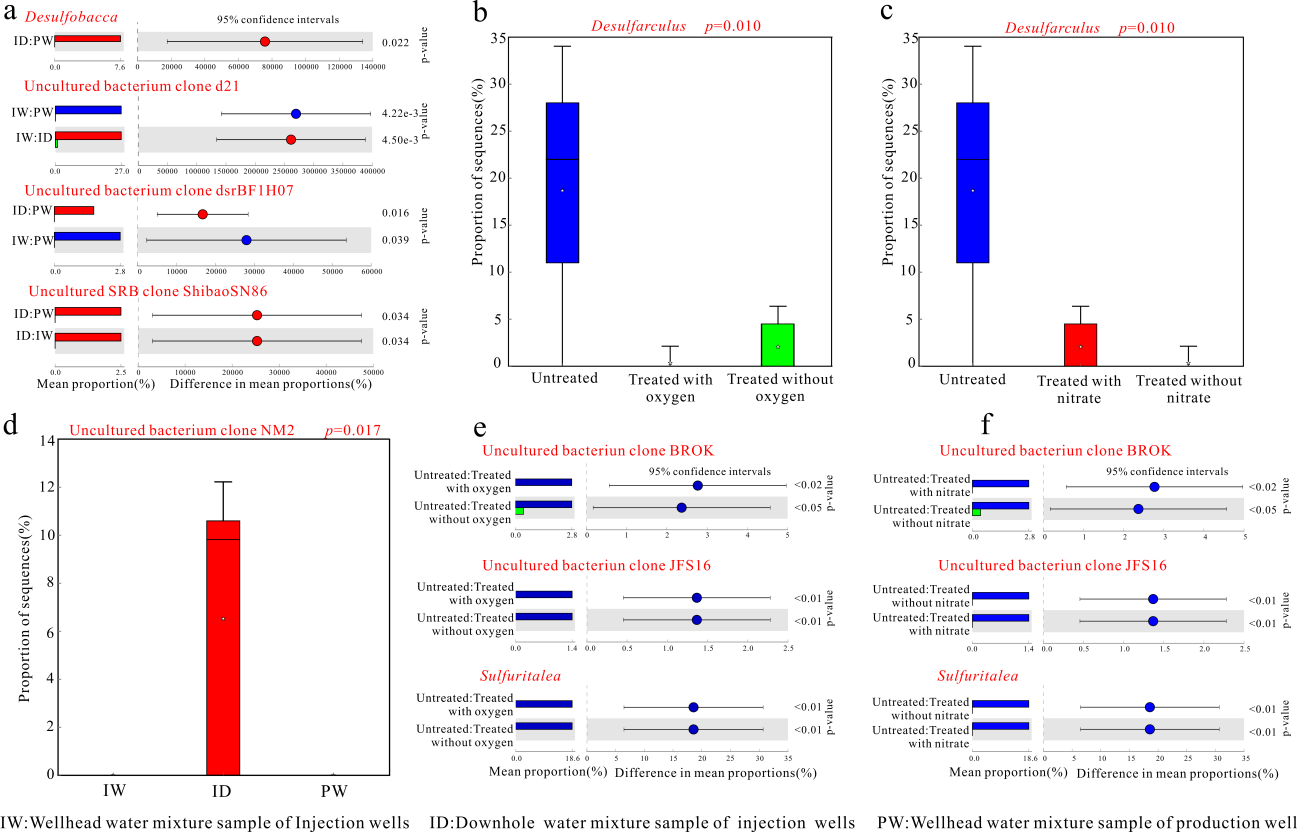


Fig. S3 Difference analysis of SRB (a-c) and SOB (d-f) genera among multiple samples at 95% confidence intervals. a, d: different groups; b, e: different oxygen levels; c, f: different nitrate levels.


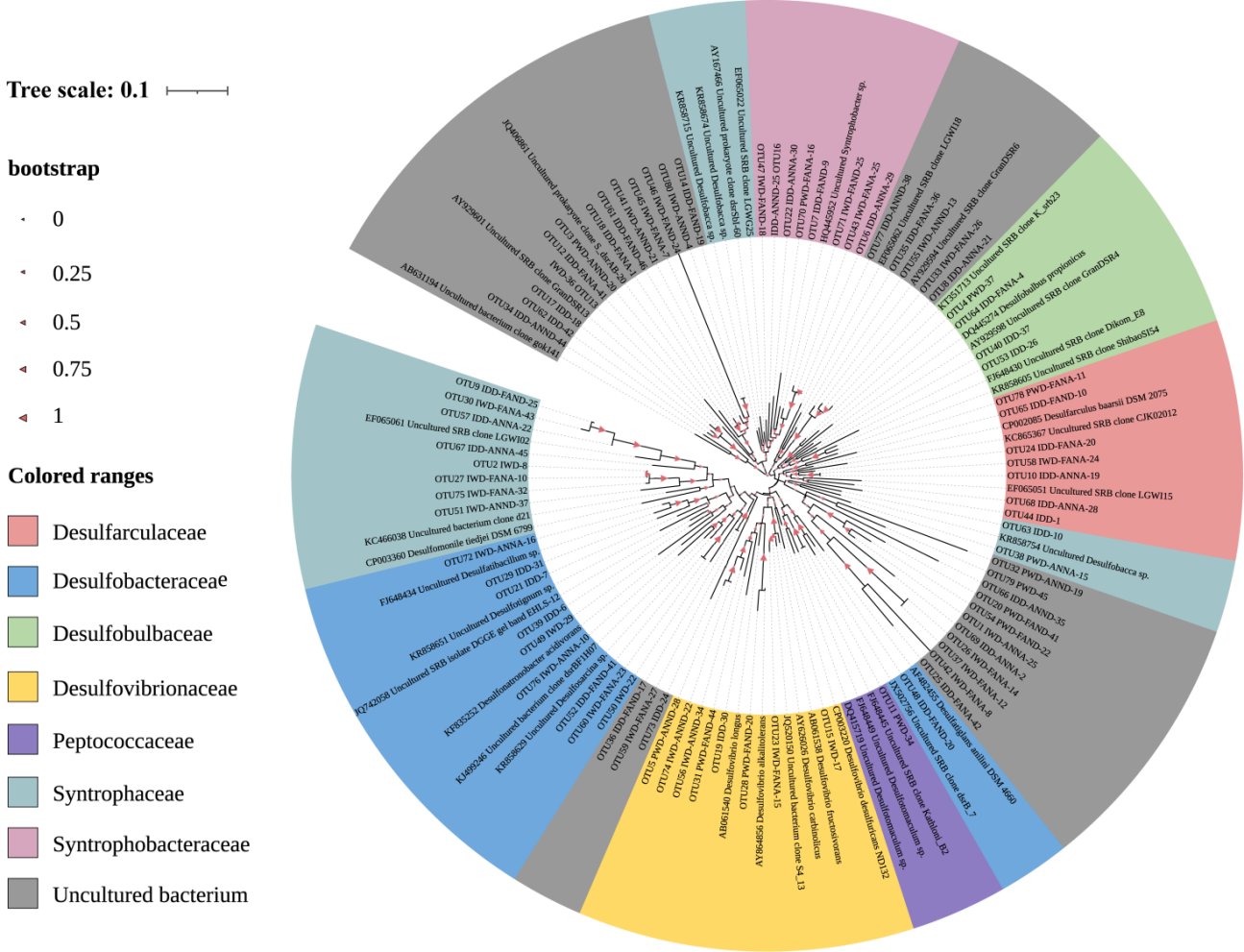


Fig. S4 Phylogenetic tree of *dsrB* genes constructed by the neighbour-joining method with 1000 bootstrap replicates at the genus level. The phylogenetic analyses were carried out by using representative sequences for each OTU and blasted with the GenBank to identify their phylogenetic affiliations. All the aligned sequences used in this tree were obtained from the NCBI GenBank database. For 80 representative sequences, they were affiliated to seven defined families including Desulfarculaceae, Desulfobacteraceae, Desulfobulbaceae, Desulfovibrionaceae, Peptococcaceae, Syntrophaceae, Syntrophobacteraceae and undefined family.


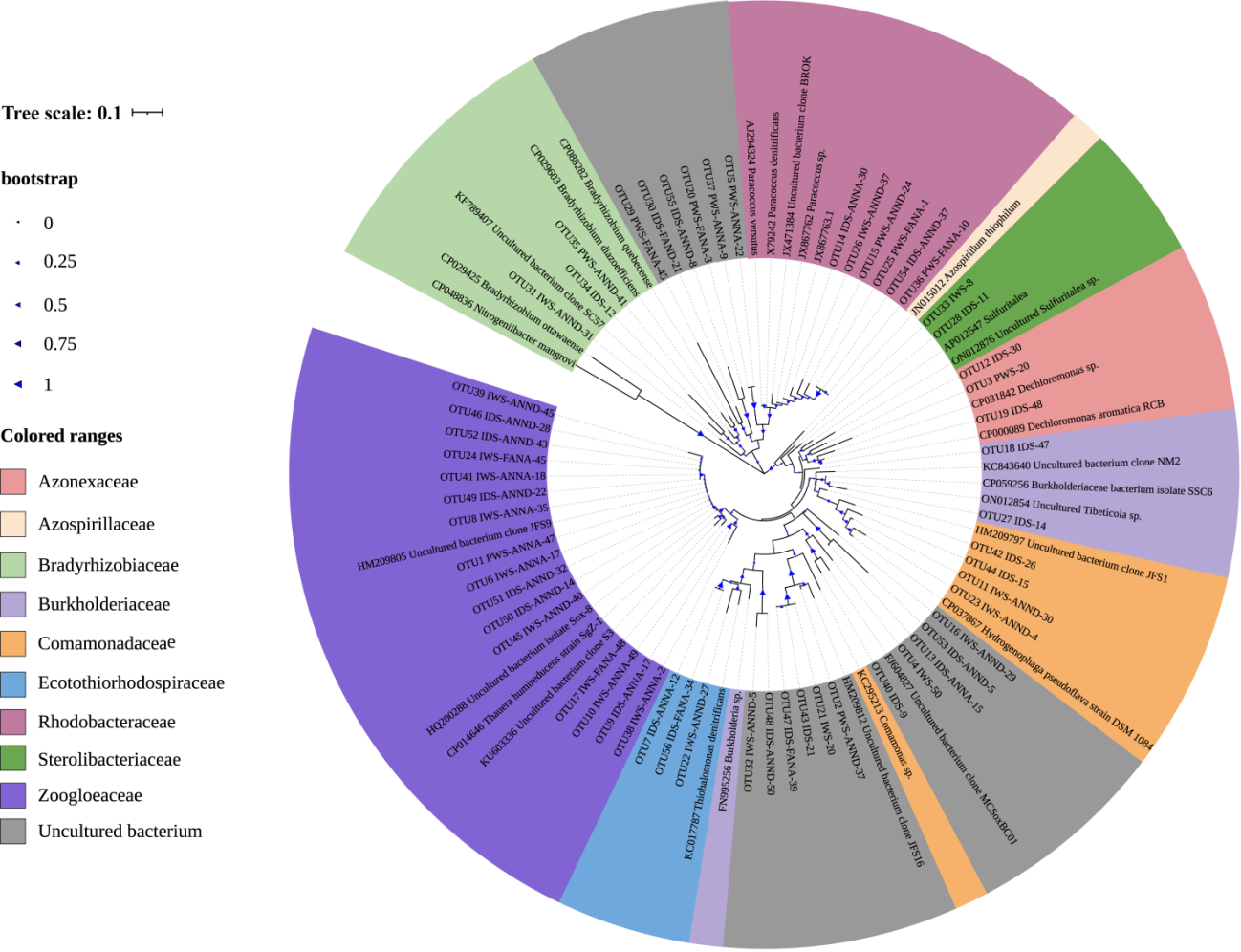


Fig. S5 Phylogenetic tree of *soxB* genes constructed by the neighbour-joining method with 1000 bootstrap replicates at the genus level. The phylogenetic analyses were carried out by using representative sequences for each OTU selected using Mothur software and they were blasted with the GenBank to identify their phylogenetic affiliations. All the aligned sequences used in this tree were obtained from the NCBI GenBank database. For 56 representative sequences, they were affiliated with nine defined families including Azospirillaceae, Bradyrhizobiaceae, Azonexaceae, Comamonadaceae, Rhodobacteraceae, Burkholderiaceae, Sterolibacteriaceae, Zoogloeaceae, Ectothiorhodospiraceae

and undefined family.


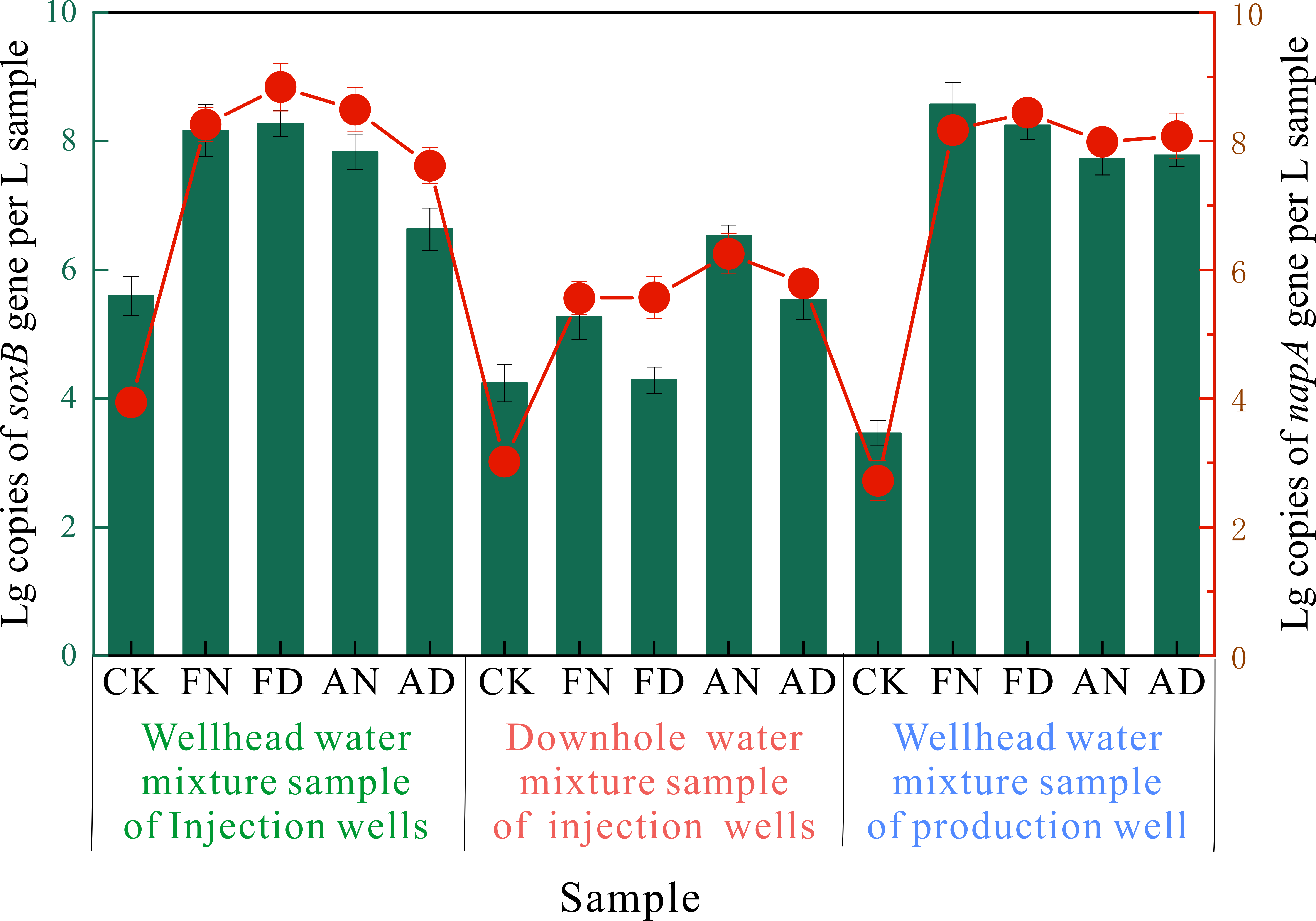


Fig. S6 Comparison of copy numbers of *soxB* and *napA* genes for 15 samples.
